# Live Biotherapeutic Zowell Reprograms Microbiota–Lipid Crosstalk to Enhance Cisplatin Efficacy in Lung Cancer

**DOI:** 10.3390/cancers18152447

**Published:** 2026-07-30

**Authors:** Bobban Subhadra, Ryan Green, Prakasha Kempaiah, Niya Bobban, Lary A. Robinson, Subhra Mohapatra

**Affiliations:** 1 Biom Pharmaceutical Corporation, Sarasota, FL 34234, USA; prakasha@biompharma.com (P.K.); niya@biompharma.com (N.B.); 2 Department of Molecular Medicine, Morsani College of Medicine, University of South Florida, Tampa, FL 33612, USA; rjgreen@usf.edu; 3 Research Service, James A. Haley Veterans Hospital, Tampa, FL 33612, USA; 4 Division of Thoracic Oncology, Moffitt Cancer Center, Tampa, FL 33612, USA; lary.robinson@moffitt.org

**Keywords:** lung cancer, gut microbiome, cancer therapy, cisplatin, live bacterial therapeutic (LBT)

## Abstract

Background: Lung cancer (LC) remains a leading cause of cancer-related mortality, often compounded by suboptimal chemotherapy efficacy and systemic toxicity. Emerging evidence implicates the gut microbiota in modulating tumor progression, immune function, and treatment outcomes. Methods: We evaluated Zowell, a novel live bacterial therapeutic (LBT) developed via LiveBiom^®^ co-fermentation, as an adjunct to cisplatin in a murine LC model harboring humanized microbiota. Mice were assigned to treatment groups: Zowell alone, cisplatin alone, their combination, and fecal microbiota transplantation (FMT) as a benchmark. Results: Zowell monotherapy significantly reduced tumor burden, and its combination with cisplatin produced synergistic anti-tumor effects. 16S rRNA sequencing revealed enrichment of beneficial taxa (*Bifidobacterium*, *Lactobacillus*, and *Allobaculum*) and suppression of contextual genera (*Clostridium* and *Akkermansia*). Treatment rebalanced gut ecology, evidenced by a lowered Firmicutes/Bacteroidetes ratio and increased alpha diversity. Zowell outperformed FMT in reducing tumor volume and inflammatory indices. Lipidomic profiling of tumor tissues identified elevated levels of immunomodulatory lipid mediators, including resolvins and prostanoids, suggesting remodeling of the tumor microenvironment toward inflammation resolution and immune activation. Conclusions: These findings support Zowell as a precision microbiome therapeutic that potentiates chemotherapy through gut microbial reprogramming and tumor lipid signaling modulation, offering translational promise for enhancing immunotherapeutic response and mitigating chemotherapy-induced toxicity.

## Simple Summary

Strategies to improve chemotherapy regimens in lung cancer remain a dire unmet clinical need. Herein, we hypothesized that manipulating the gut microbiome may improve lung cancer outcomes through alterations in immune and inflammatory mediators via the gut–lung axis. We therefore developed “Zowell”, a novel live bacterial therapeutics, and found it to reduce lung cancer growth in a mouse model when compared to both no treatment and to standard fecal microbiota transfer therapy. Furthermore, Zowell acted synergistically with the chemotherapeutic cisplatin and improved several metrics of gut health, including microbiome diversity and anti-inflammatory lipid mediators.

## 1. Introduction

Lung cancer (LC) remains a leading cause of cancer-related mortality, with lung adenocarcinoma (LUAD), squamous cell lung cancer (SCC), and small cell lung cancer (SCLC) comprising over 90% of cases [1,2,3]. Globally, LC is the most commonly diagnosed cancer and the leading cause of cancer-related death overall in men worldwide, with almost 2.5 million cases and 1.8 million deaths. Gut dysbiosis affects 70–75% of cancer patients, reducing treatment tolerance, increasing hospitalization, and lowering survival rates [4,5,6]. Emerging research underscores the gut microbiome’s role in LC progression and treatment response by modulating inflammation, DNA damage, and metabolite production [7,8]. Gut dysbiosis in LC is characterized by reduced butyrate-producing bacteria, increased pro-inflammatory pathogens, and altered metabolite production [9,10,11]. Specific microbial signatures influence immune checkpoint inhibitor (ICI) efficacy [12], while preclinical studies demonstrate that microbiome modulation enhances PD-1 blockade responses [13,14]. Additionally, high-fiber diets, polyphenols, and probiotics have shown promise in improving cancer treatment outcomes [9].

The gut microbiota regulates LC susceptibility and progression via inflammation modulation, DNA damage repair, and tumor-associated metabolite production [7,8]. Dysbiosis in LC patients includes a decrease in beneficial commensals, such as butyrate-producing bacteria, and an increase in pro-inflammatory opportunistic pathogens [9,10,11]. LC is also characterized by altered bacterial metabolite production, including short-chain fatty acids (SCFAs) and polyamines, which are directly involved in cancer metabolism. Using “omics” approaches, researchers have identified gut microbiota signatures that predict treatment responses, stratifying patients as responders vs. non-responders based on the presence of commensal genera and species such as Clostridiales, Ruminococcaceae, *Faecalibacterium* spp., *Akkermansia muciniphila*, *Bacteroides fragilis*, Bifidobacteria, Enterococci, Collinsella, and Alistipes [12,15,16,17]. Live bacterial therapeutics (LBTs) targeting gut homeostasis have been explored to enhance immunotherapy efficacy. Despite advances in cancer therapy, microbiome-based interventions remain underutilized. In this context, the ability to monitor and condition gut microbiota could optimize LC treatment efficacy, reduce side effects, and improve progression-free survival.

The gut microbiome is increasingly recognized as a key modulator of cancer progression and therapeutic response. However, translating this knowledge into actionable interventions remains a challenge. This study introduces Zowell, a rationally designed live bacterial therapeutic (LBT) to reshape the gut microbiota [18]. We hypothesize that Zowell treatment will reshape the gut microbiota profile and, thus, create a tumor microenvironment that is more restrictive to tumor growth via soluble and immune mediators, such as SCFAs and bioactive lipids. We aim to determine whether these dual effects, microbial and lipidomic, synergize with cisplatin to suppress tumor growth more effectively than chemotherapy alone. For this, we will compare the effects of Zowell to those of a standard and clinically approved fecal microbiota transplantation (FMT) formulation to determine any advantage of targeted microbiome modulation (Zowell) over broad-spectrum approaches (FMT). Our findings will provide mechanistic insight into how microbiome-based therapeutics can be leveraged to improve cancer outcomes and support the integration of precision microbiome modulation into standard oncology care.

Biom Pharmaceuticals is at the forefront of precision microbiome-based adjunct biologics, leveraging LiveBiom^®^ technology to develop gut-adapted, immune-modulating therapies such as Zowell [18,19,20,21]. Zowell, developed by Biom Pharmaceuticals, is a co-fermentation-based microbiome-modulating biologic using LiveBiom^®^ technology [22]. This patented fermentation process enables the growth of LBTs on an immobilized substrate with prebiotics and metabolites, generating gut-adapted bioactive compounds. The selected probiotic strains exhibit SCFA production, mucin degradation, and tight junction-strengthening properties, while their exopolysaccharide (EPS) production enhances SCFA microbial formation. Our primary objective in this study was to determine whether Zowell, as an adjunct biologic therapy, improves the chemotherapy (cisplatin) in an LC mouse model.

## 2. Materials and Methods

**Figure 1:**
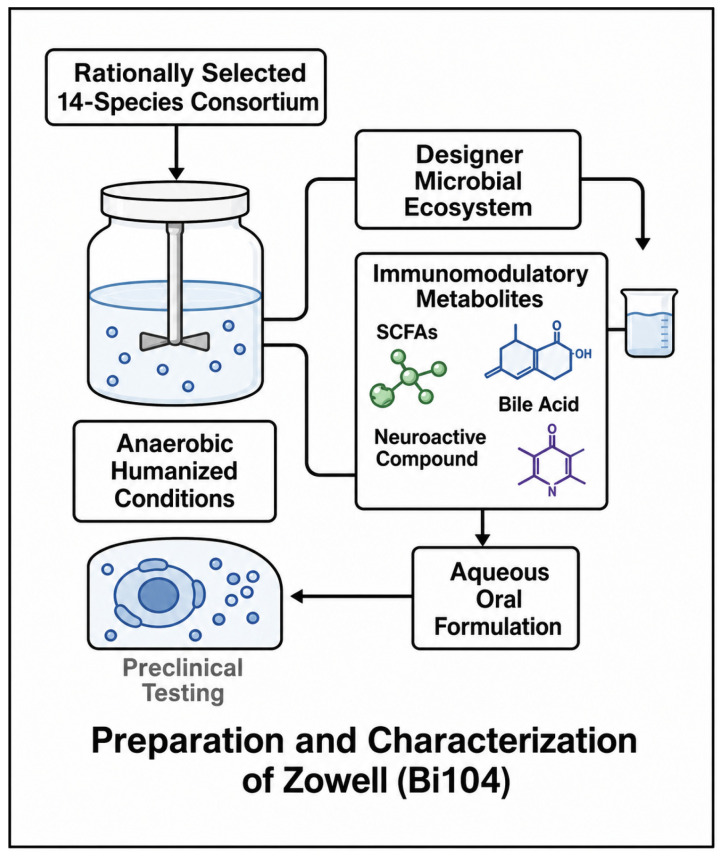
Flow-chart of LiveBiom fermentation technology used for production of Zowell. Zowell was produced using Biom Pharmaceutical’s patented LiveBiom^®^ co-fermentation technology, which replicates anaerobic, gut-mimicking culture conditions to support stable microbial colonization and bioactive metabolite production.

Preparation and Characterization of Zowell: Zowell is a biologic live bacterial therapeutic (LBT) designed to modulate the gut microbiome in oncology settings. It comprises a rationally selected 14-species consortium of human commensal bacteria, formulated with immunonutrient-rich prebiotics and a proprietary postbiotic matrix. The consortium consists of carefully curated strains of *Lactobacillus johnsonii*, *Bifidobacterium animalis*, *Lentilactobacillus kefiri*, *Clostridium butyricum*, and *Bifidobacterium bifidum*, together with *Lacticaseibacillus rhamnosus*, *Bifidobacterium longum*, *Lactobacillus acidophilus*, *Lactiplantibacillus plantarum*, *Limosilactobacillus fermentum*, *Limosilactobacillus reuteri*, *Lacticaseibacillus casei*, *Bacillus coagulans*, and *Lactobacillus crispatus*. Genus assignments follow the 2020 reclassification of the former genus *Lactobacillus*; the species previously designated *Lactobacillus kefiri*, *L. rhamnosus*, *L. plantarum*, *L. fermentum*, *L. reuteri*, and *L. casei* are now classified within the genera *Lentilactobacillus*, *Lacticaseibacillus*, *Lactiplantibacillus*, and *Limosilactobacillus*, respectively. The formulation was developed using Biom Pharmaceutical (Sarasota, FL, USA)’s patented LiveBiom^®^ co-fermentation technology, which replicates anaerobic, gut-mimicking culture conditions to support stable microbial colonization and bioactive metabolite production (Figure 1). These conditions, carefully calibrated for physiological pH, redox potential, osmolarity, and nutrient gradients, enable strain adaptation and enhance the fidelity of microbiota–host interactions.

The 14 strains and their specific proportions were selected based on their expanded genetic repertoires, metabolic versatility, and functional roles in immune modulation, including short-chain fatty acid (SCFA) biosynthesis, secondary bile acid conversion, and neurotransmitter analog secretion. These taxa were cultivated in parallel using a proprietary bioreactor system that facilitates interspecies metabolic crosstalk, optimizing community resilience and functional redundancy within the therapeutic ecosystem.

The final oral formulation, designated Bi104, is an aqueous, ingestible preparation incorporating GRAS-status (Generally Recognized as Safe) prebiotic and postbiotic components with well-established safety profiles derived from previously validated consumer health products by Biom Pharmaceuticals. Scalable GMP-compliant manufacturing protocols were developed in-house to ensure batch-to-batch reproducibility, strain viability, and consistent therapeutic potency. Rigorous quality control measures—including 16S rRNA sequencing, metabolomic profiling, and potency assays—are integrated into the production workflow.

Zowell was produced at kilogram-scale under standardized manufacturing conditions, and its composition and bioactivity are described extensively in prior published studies [18,19,20]. This formulation underpins the current study, investigating its efficacy in modulating gut microbial ecology and enhancing cisplatin-based chemotherapy in lung cancer [18,19,20].

**Figure 2:**
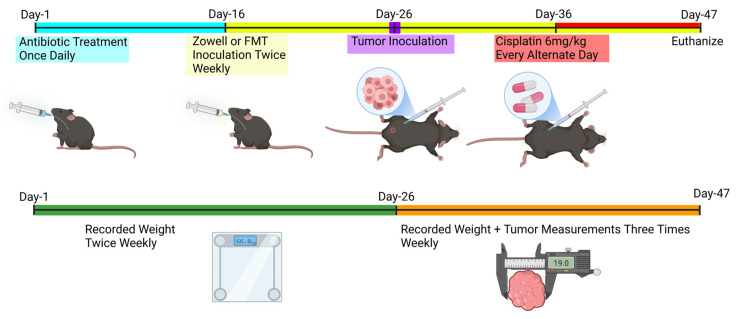
Experimental outline: Thirty C57Bl/6 mice were treated daily with an antibiotic cocktail by oral gavage (vancomycin 50 mg/kg, ampicillin 100 mg/kg, metronidazole 100 mg/kg) for a duration of two weeks. The mice were randomly assigned to six treatment groups (*n* = 5 per group): vehicle, cisplatin alone, Zowell, Zowell + cisplatin, FMT, and FMT + cisplatin. Following the completion of the antibiotic treatment, vehicle, FMT, or Zowell was administered by oral gavage daily. The administration of vehicle, FMT, or Zowell was continued for the duration of the trial. On day 26, subcutaneous flank tumors were initiated by injection of 1 × 10^6^ LLC cells into the right hind flank. Treatment with 6 mg/kg cisplatin IP every other day was started on day 36 and continued every other day. Fecal pellets were collected and snap-frozen pre-tumor initiation, 1 week post-tumor initiation, and at endpoint. Body weights and tumor measurements were recorded as shown.

Study Design and Animal Model: This study aimed to evaluate the efficacy of Zowell as an adjunct to cisplatin chemotherapy in a preclinical murine model of lung cancer, with a focus on tumor reduction and gut microbiome modulation. As illustrated in Figure 2, thirty C57BL/6 mice underwent microbiota depletion using a broad-spectrum oral antibiotic cocktail (vancomycin 50 mg/kg, ampicillin 100 mg/kg, and metronidazole 100 mg/kg, Thermo Scientific Chemicals, Ward Hill MA, USA) administered daily for 14 days. To establish a humanized gut microbial landscape conducive to colonization and immunometabolic responsiveness, mice subsequently received either Zowell, investigational fecal microbiota transplantation (FMT; OpenBiome: Cambridge MA, USA), or vehicle control via oral gavage. OpenBiome provides fecal microbiota transplantation (FMT) material from extensively screened healthy human donors for clinical and research use. Thus, the FMT obtained from OpenBiome is a complex mixture, containing the complete community of microorganisms found in the donor’s gut.

Following microbiota modulation, animals were randomized into six treatment groups (*n* = 5 per group): 1. Vehicle control; 2. Cisplatin alone (Tocris Bioscience Minneapolis MN, USA); 3. Zowell alone; 4. Zowell + cisplatin; 5. FMT alone; 6. FMT + cisplatin. Zowell (5 × 10^8^ CFU in 200 µL of PBS) or vehicle was administered orally on a daily schedule, beginning on study day 16. The dose was selected based on previous in-house lab studies based on viability. Subcutaneous flank tumors were established on day 26 by injection of 1 × 10^6^ Lewis Lung Carcinoma (LLC, ATCC Manassas VA, USA) cells into the right hind flank. Cisplatin treatment (intraperitoneal injection, 6 mg/kg) commenced on day 36 and was administered every other day.

Throughout the study duration, body weight and tumor volume were monitored using standard caliper measurements. Fecal samples were collected at three defined time points—pre-tumor implantation (baseline), pre-cisplatin administration, and endpoint—to enable comprehensive metagenomic analysis of microbiota structure and dynamics.

Primary endpoints included tumor volume reduction and microbiota composition shifts; secondary endpoints encompassed body weight trends, alpha-/beta-diversity indices, and differential abundance of taxa linked to anticancer immunity and metabolic resilience. All experimental procedures were conducted under protocols approved by the institutional animal care committee.

Lung Cancer Microbiome Animal Model: To investigate the efficacy of Zowell and its potential synergy with cisplatin chemotherapy, we employed a syngeneic Lewis Lung Carcinoma (LLC) subcutaneous flank tumor model in C57BL/6 mice. A total of 30 mice were administered a broad-spectrum antibiotic cocktail via oral gavage, vancomycin (50 mg/kg), ampicillin (100 mg/kg), and metronidazole (100 mg/kg), once daily for two weeks to disrupt native gut microbiota and facilitate probiotic colonization. Following cessation of antibiotic treatment, mice received one of three microbiota restoration interventions via oral gavage: Zowell (a defined live bacterial therapeutic), fecal microbiota transplantation (FMT; OpenBiome), or vehicle control.

Animals were randomly assigned to six treatment groups (*n* = 5 per group), housed in pairs of cages per group. Zowell and FMT formulations or vehicle were delivered daily via oral gavage (200 µL of 5 × 10^8^ CFU in PBS). On day 26, all mice were inoculated with 1 × 10^6^ LLC cells subcutaneously into the right hind flank to induce tumor formation. Tumor dimensions and body weights were recorded biweekly using caliper measurements and standardized weighing procedures. Following completion of the treatment protocol, animals were humanely sacrificed in accordance with institutional ethical guidelines. Collected tumor tissues were reserved for downstream lipidomic profiling, while fecal samples were obtained at designated time points for microbiome analysis. This design allowed comparative evaluation of Zowell versus FMT and vehicle controls, with integrated assessment of tumor response and host microbial ecology.

Fecal Sample Collection and Microbiome Analysis: Fecal samples were collected at three defined time points: one day prior to tumor implantation (baseline), one week post-tumor initiation, and at the study endpoint. Samples were snap-frozen and stored at −80 °C until processing. Microbial DNA was extracted using a modified protocol based on the Zymo DNA Fecal/Soil Extraction Kit, following previously published methods. DNA quality and concentration were verified prior to sequencing.

Microbiome profiling was performed using 16S rRNA gene sequencing on the Illumina MiSeq platform. Amplicons targeting the V3–V4 hypervariable regions were generated and aligned to reference databases to identify microbial taxa, clustered into operational taxonomic units (OTUs) based on sequence similarity. Bioinformatic analysis was conducted using QIIME 2 (Ver. 2024.10), with standard pipelines applied for taxonomic classification, diversity metrics, and community structure assessment. Alpha diversity was calculated using multiple indices, including OTU count, Shannon index, and Simpson index, to quantify within-sample richness and evenness. Beta-diversity analyses were performed to assess between-group differences in microbial composition. Relative abundances of taxa with known pro-inflammatory or anti-inflammatory roles were compared across treatment groups.

Functional annotation was conducted using KEGG pathway mapping and module tables to infer metabolic potential. All primary data processing and annotation were performed by the USF Center for Microbiome Research Core Facility. Taxa with established biomarker relevance in lung cancer were specifically evaluated to determine treatment-associated shifts in microbial ecology.

Tumor Lipid Extraction and Targeted Lipidomics: Lipids were extracted from LLC flank tumor tissues harvested from vehicle- and treatment-group mice. Tissues were homogenized and subjected to methanol–chloroform extraction using standard protocols to isolate total lipids. A targeted lipidomics approach was employed, focusing on a curated panel of 55 lipids selected based on structural characteristics and reported bioactivity.

Quantitative analysis was performed using a Sciex (Marlborough MA, USA) QTRAP 6500+ triple-quadrupole mass spectrometer operated with Analyst software version 2.0. Chromatographic separation and mass spectrometric detection parameters were optimized for sensitivity and specificity. Data integration and quantification were conducted using Sciex OS software version 1.7. Lipid species were annotated and compared across groups to identify treatment-associated shifts in lipid profiles relevant to tumor biology and host–microbiome interactions.

Statistical Analysis: All quantitative data are presented as mean ± standard error of the mean (SEM), unless otherwise specified. Statistical comparisons between groups were performed using one-way or two-way analysis of variance (ANOVA), followed by Holm–Sidak post hoc tests for multiple comparisons. A *p*-value < 0.05 was considered statistically significant. Graphical representations were generated using Prism version 8.0 (GraphPad Software, San Diego, CA, USA). Error bars in all figures denote mean ± SEM, unless stated otherwise.

## 3. Results

Zowell Treatment Reduces Tumor Burden and Modulates Host Physiology: To establish a humanized gut microbiome, mice were depleted of native gut flora with a broad-spectrum antibiotic cocktail for fourteen days and then colonized by daily oral gavage with the live biotherapeutic Zowell, a benchmark human fecal microbiota transplant (FMT; OpenBiome), or vehicle beginning on day 16. Subcutaneous flank tumors were established on day 26 (1 × 10^6^ LLC cells), and within each microbiome condition, mice were treated with or without cisplatin from day 36, yielding six groups (*n* = 5): vehicle, cisplatin alone, Zowell, Zowell + cisplatin, FMT, and FMT + cisplatin (Figure 2).

**Figure 3:**
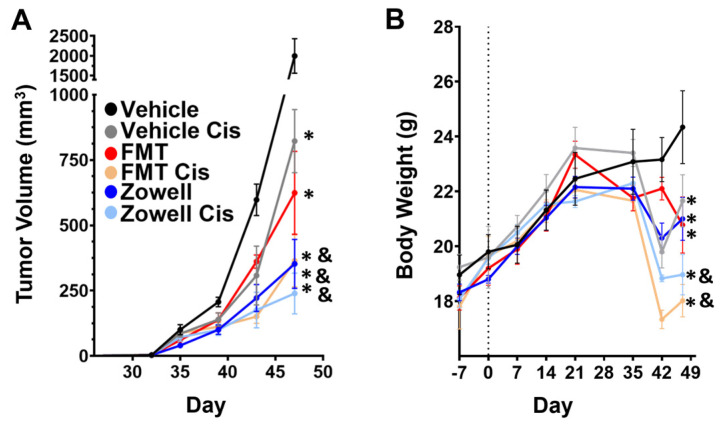
Microbiome targeted therapies are effective with or without cisplatin chemotherapy. Zowell treatment shows a reduction in tumor size in the LLC mouse model. (**A**) Tumor volume: Zowell independently reduces tumor burden and potentiates cisplatin efficacy. (**B**) Body weight: Zowell or FMT alone caused minimal change in body weight, while cisplatin groups experienced significant weight loss. Cis = cisplatin, * = vs. vehicle, & = vs. vehicle Cis *n* = 5, *p* ≤ 0.05.

Zowell monotherapy significantly reduced tumor volume compared to vehicle-treated controls (Figure 3A). Notably, the combination of Zowell with cisplatin produced a synergistic effect, resulting in greater tumor suppression than cisplatin alone. FMT also conferred a protective effect, reducing tumor growth relative to antibiotic-treated mice without recolonization, although the magnitude of reduction was less pronounced than with Zowell treatment.

Body weight analysis revealed cisplatin-associated weight loss across all treatment groups (Figure 3B). In mice lacking microbial recolonization, this effect was partially obscured by increased tumor burden. Conversely, mice receiving FMT or Zowell exhibited lower endpoint body weights relative to vehicle controls, attributable in part to a reduced tumor mass in treated groups. Statistical comparisons were performed using ANOVA with Holm–Sidak correction (*p* ≤ 0.05).

Zowell Modulates Gut Microbiota Composition and Diversity: To investigate microbial changes underlying the observed differences in tumor growth, we performed metagenomic analysis of fecal samples collected at three time points: pre-tumor implantation, post-tumor initiation, and at study endpoint. Sequencing was conducted individually for each time point, followed by a pooled analysis to assess treatment-specific effects independent of tumor progression.

**Figure 4:**
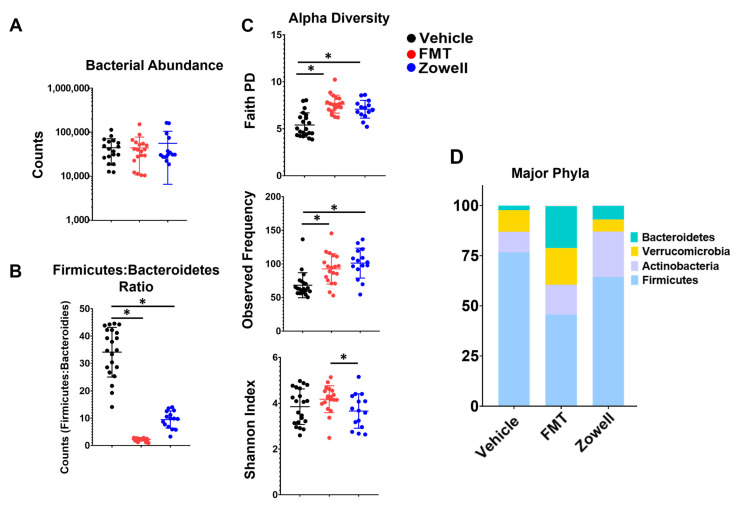
Zowell or FMT alter alpha diversity without changing total bacterial abundance: (**A**) Total bacterial abundance as measured by 16s rRNA counts. (**B**) Ratio of counts representing Firmicutes vs. Bacteroidetes phyla. (**C**) Alpha diversity of the samples calculated using Faith’s PD, observed frequency, and Shannon methods. (**D**) Composition of the microbiome at the phyla level. N = 21 V, 19FMT, 14Z * = *p* ≤ 0.05.

Total bacterial abundance was comparable across groups, averaging ~40,000 reads per sample (Figure 4A). Although the Zowell group trended toward higher abundance, differences were not statistically significant. Taxonomic profiling at the phylum level revealed shifts in the Firmicutes/Bacteroidetes (F/B) ratio, a key indicator of gut homeostasis. Both Zowell and FMT treatments significantly reduced the F/B ratio compared to vehicle controls (Figure 4B), consistent with probiotic-mediated restoration of microbial balance. Notably, the F/B ratio remained higher in Zowell-treated mice than in the FMT group, potentially reflecting its defined microbial composition.

Alpha diversity was assessed using Faith’s phylogenetic diversity, observed features, and the Shannon index (Figure 4C). Zowell and FMT both increased alpha diversity relative to the vehicle group, with FMT showing slightly higher diversity by the Shannon index. Dominant phyla included Firmicutes, Actinobacteria, and Verrucomicrobia, collectively comprising 80–100% of detected taxa (Figure 4D). Bacteroidetes abundance increased in both treatment groups, more prominently in FMT-treated mice.

**Figure 5:**
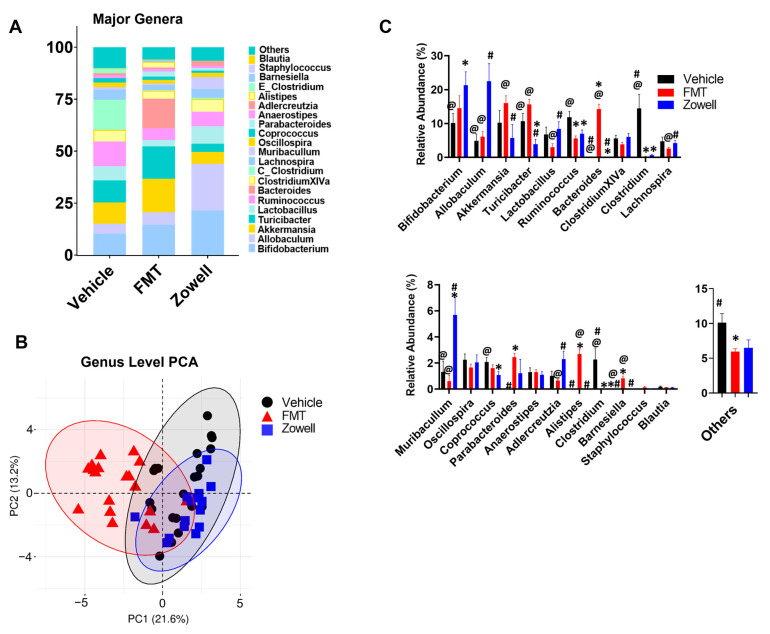
Zowell or FMT alter gut β-diversity: (**A**) Composition of the microbiome of vehicle-, FMT-, or Zowell-treated mice at the genus level. (**B**) PCA analysis of relative genera abundance values. (**C**) Significant changes in relative abundance of genera between groups. N = 21 V, 19FMT, 14Z *, @, or # = *p* ≤ 0.05, * = vs. vehicle, @ = vs. FMT, # = vs. Zowell.

Beta-diversity analysis revealed treatment-specific clustering at the genus level (Figure 5A). Principal component analysis (PCA) showed that FMT-treated microbiomes were more distinct from both the vehicle and Zowell groups (Figure 5B), likely due to the broader microbial spectrum introduced via donor stool. Zowell-treated mice clustered closer to the vehicle group, reflecting its targeted probiotic composition.

**Figure 6:**
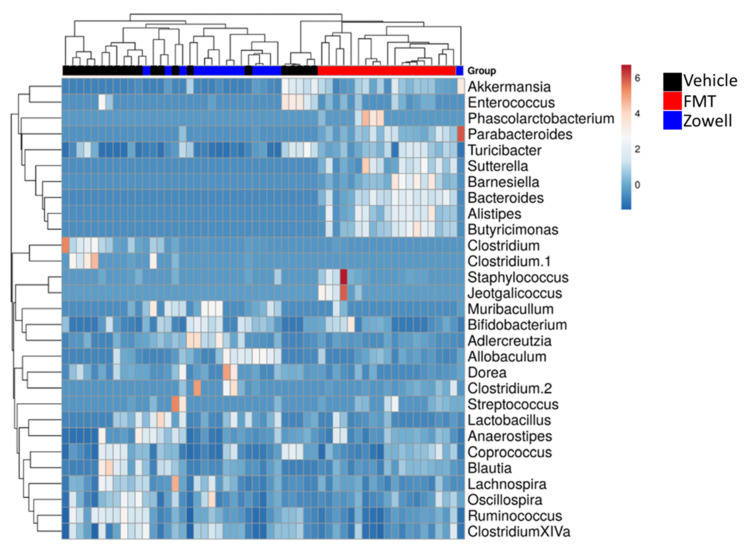
Heatmap of relative abundance of major genera reveals within-group similarities. Unit variance scaling is applied to rows. Both rows and columns are clustered using correlation distance and average linkage. Colors indicate normalized relative abundance.

At the family level, Zowell treatment enriched Bifidobacteriaceae and Erysipelotrichaceae, while reducing Verrucomicrobiaceae and Turicibacteraceae. Genus-level analysis showed increased abundance of Bifidobacterium, Allobaculum, Lactobacillus, Muribaculum, and Adlercreutzia, alongside reduced levels of Akkermansia, Turicibacter, Ruminococcus, Clostridium, and Coprococcus (Figure 5C). FMT also elevated Bifidobacterium and Akkermansia but differed significantly from Zowell in the abundance of Turicibacter, Lactobacillus, Bacteroides, Allobaculum, Muribaculum, Alistipes, and Barnesiella. Intra- and inter-group microbial distributions were visualized via heatmap analysis (Figure 6), highlighting distinct compositional signatures associated with each treatment. Notably, unbiased clustering of the genus-level microbial abundance data yielded high within-group similarity between samples.

Zowell Reprograms the Tumor Lipidome Toward Inflammation Resolution and Immune Activation: To elucidate the immunometabolic effects of Zowell treatment, we performed targeted lipidomic profiling of tumor tissues using a curated panel of 55 immunoregulatory lipids. Mass spectrometry analysis revealed that Zowell-treated tumors exhibited alterations in lipid abundance compared to vehicle controls, with 21 lipid species showing a fold change ≥2.

**Figure 7:**
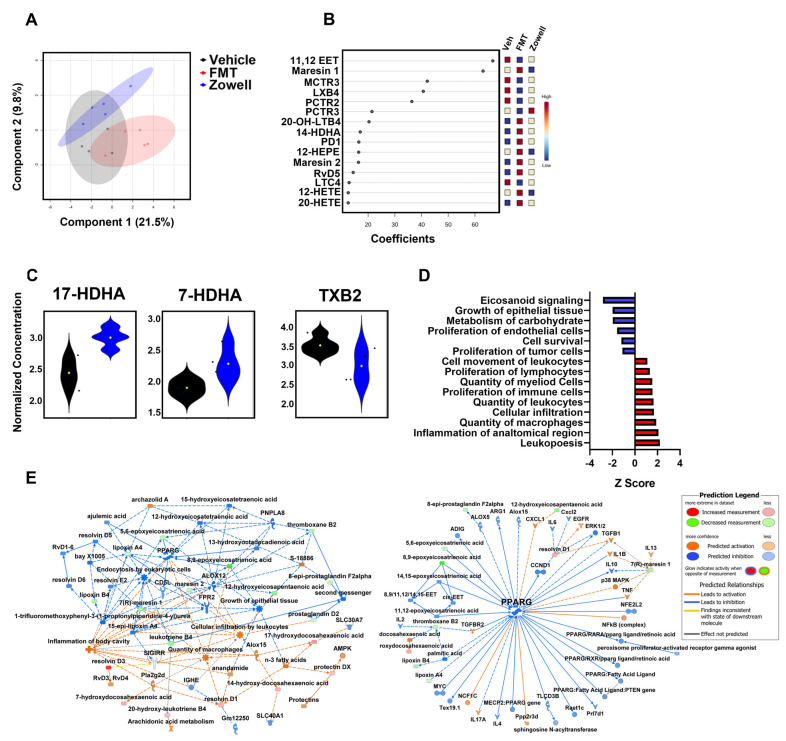
Zowell alters relative abundance of lipids within tumors: (**A**) Partial Least Squares Discriminant Analysis of lipid abundance data. (**B**) The weighted sum of absolute regression coefficients calculated by the PLS-DA. The colored boxes on the right indicate the relative concentrations of lipids. The coefficient indicates the importance of each lipid to the PLS-DA. (**C**) Violin plots depicting the three most significantly altered lipids in the Zowell vs. vehicle comparison (Student’s *t*-test). (**D**) Pathway analysis (IPA) was used to determine the top cellular functions (ranked by pathway Z-score) altered by Zowell given the lipid abundance data. (**E**) Gene and function activity networks (IPA) showing predicted activation or inhibition of genes/functions based on the lipid abundance data (left: general network of genes directly connected to the panel lipids; right: interactions with the PPAR gamma hub gene).

Partial Least Squares Discriminant Analysis (PLS-DA) demonstrated distinct clustering of Zowell and FMT treatment groups, indicating divergent lipidomic signatures (Figure 7A). PLS-DA coefficient analysis identified key lipids driving this separation, including 11,12-EET, Maresin 1, MCTR3, Lipoxin B4 (LXB4), and Protectin CTR2 (PCTR2) (Figure 7B). Among these, 17-HDHA, 7-HDHA, and thromboxane B2 (TBX2) exhibited the most statistically significant differences between the Zowell and vehicle groups (Figure 7C). Ingenuity Pathway Analysis (IPA) of the lipidomic dataset revealed enrichment of signaling pathways associated with eicosanoid biosynthesis, endothelial cell proliferation, and leukopoiesis (Figure 7D,E). Functional network mapping predicted activation of leukocyte infiltration and macrophage recruitment, alongside inhibition of the nuclear receptor PPAR-γ, a key regulator of lipid metabolism and immune suppression.

These findings suggest that Zowell not only modulates gut microbial composition but also reprograms tumor-associated lipid signaling, promoting a shift toward inflammation resolution and enhanced immune surveillance. This dual microbiome–lipid axis may underlie the observed synergy with cisplatin and supports the therapeutic potential of Zowell in lung cancer.

## 4. Discussion

Our study demonstrates that Zowell, a rationally designed microbiome-modulating live bacterial therapeutic (LBT), significantly enhances cisplatin efficacy in a preclinical lung cancer (LC) model. Zowell monotherapy reduced tumor burden, and its combination with cisplatin yielded synergistic tumor suppression, surpassing cisplatin alone. These findings align with emerging evidence implicating the gut microbiome as a critical regulator of cancer progression and therapeutic responsiveness [7,8,9].

Zowell administration reshaped the gut microbial landscape, increasing the abundance of beneficial genera, such as Bifidobacterium, Lactobacillus, Allobaculum, and Adlercreutzia, while reducing pathogenic taxa, including Akkermansia and Clostridium. These shifts are consistent with prior reports linking specific commensals to enhanced anti-tumor immunity and improved outcomes with chemotherapy and immune checkpoint blockade [12,13,14]. Notably, Zowell reduced the Firmicutes/Bacteroidetes (F/B) ratio, a marker of gut homeostasis and increased alpha-diversity metrics (Faith’s PD, observed features), both of which are associated with reduced systemic inflammation and improved host resilience [9,23]. Prior studies have linked lower F/B ratios to reduced systemic inflammation, a crucial factor influencing cancer outcomes [9]. The observed increases in alpha diversity among Zowell-treated mice across Faith’s PD and observed feature metrics further support the notion that greater microbial diversity enhances host resilience and therapeutic responsiveness [10,11].

Microbial taxa enriched by Zowell, particularly Bifidobacterium and Lactobacillus, have been shown to augment CD8^+^ T-cell responses and potentiate anti-PD-1 therapy [14,15,16,17]. Additionally, increased Allobaculum and Muribaculum may elevate short-chain fatty acid (SCFA) production, notably butyrate, which modulates T-cell metabolism, reinforces epithelial barrier integrity, and exerts systemic anti-inflammatory effects [13,14,24]. These microbial and metabolic changes likely contribute to the enhanced cisplatin efficacy observed in our model.

The combination of Zowell and cisplatin was particularly effective, suggesting potential synergy between gut microbiome modulation and chemotherapy. Importantly, chemotherapy is known to disrupt gut microbial communities, leading to mucosal injury and systemic inflammation [25,26]. Zowell’s ability to restore beneficial taxa and boost SCFA production may mitigate chemotherapy-induced dysbiosis, reduce off-target toxicity, and foster a tumor-suppressive immune milieu [25,27]. This dual action—microbiome restoration and immune potentiation—positions Zowell as a promising adjunct to conventional chemotherapy.

Beyond microbial modulation, Zowell induced distinct alterations in the tumor lipidome, revealing systemic immunometabolic reprogramming. Lipidomic profiling identified elevated levels of specialized pro-resolving mediators (SPMs), such as Maresin 1, MCTR3, and LXB4—bioactive lipids that promote resolution of inflammation, leukocyte trafficking, and tissue repair. Ingenuity Pathway Analysis (IPA) further highlighted enrichment of eicosanoid signaling, macrophage activation, and leukopoiesis pathways, alongside predicted inhibition of PPAR-γ, a lipid-sensing nuclear receptor implicated in immune suppression. These findings suggest that Zowell modulates the tumor immune microenvironment through both microbial metabolite production and lipid signaling cascades, converging on inflammation resolution and immune activation. Similar mechanisms have been reported in probiotic and SCFA-based interventions that regulate SPM biosynthesis and eicosanoid pathways [28].

While FMT also reduced tumor burden, Zowell demonstrated superior efficacy. This may reflect its targeted enrichment for immunomodulatory and metabolically beneficial strains, in contrast to the heterogeneous microbial profiles introduced by FMT [19,23]. FMT, although it has a broad-spectrum effect, may introduce heterogeneous and less-optimized microbial profiles [14]. The precision of Zowell’s formulation likely contributes to its enhanced therapeutic performance.

### Study Limitations

Our study has limitations. Although fecal microbiome analysis revealed compositional shifts, future investigations should incorporate systemic immune profiling—including tumor-infiltrating lymphocytes (TILs), cytokine networks, and metabolomic signatures—to delineate Zowell’s mechanistic pathways. Additionally, clinical translation will require validation in more complex LC models and eventual progression to human trials.

## 5. Conclusions

This study provides compelling preclinical evidence that Zowell, a precision-engineered live bacterial therapeutic developed via LiveBiom^®^ co-fermentation technology, enhances the anti-tumor efficacy of cisplatin in a murine lung cancer model. Zowell monotherapy significantly suppressed tumor growth, and its combination with cisplatin yielded synergistic effects, surpassing chemotherapy alone. Microbiome profiling revealed that Zowell selectively enriched beneficial taxa, including Bifidobacterium, Lactobacillus, and Allobaculum, while reducing pro-inflammatory genera, such as Clostridium and Akkermansia. These shifts were associated with increased alpha diversity, a lowered Firmicutes/Bacteroidetes ratio, and improved gut ecological balance.

Compared to FMT, Zowell induced more targeted and therapeutically relevant microbial changes, likely due to its rational strain selection and metabolite-driven design. Lipidomic analysis further demonstrated that Zowell modulates tumor-associated lipid signaling, elevating specialized SPMs such as Maresin 1, LXB4, and MCTR3—lipids known to promote inflammation resolution, macrophage activation, and tissue repair. Ingenuity Pathway Analysis identified key immunometabolic pathways affected by Zowell, including eicosanoid signaling and leukocyte infiltration, alongside inhibition of PPAR-γ, a central regulator of lipid metabolism and immune suppression.

Together, these findings suggest that Zowell acts through dual microbial and lipidomic axes to reprogram the tumor microenvironment, enhance host immunity, and potentiate chemotherapy. This study supports the integration of precision microbiome therapeutics into oncology protocols as a strategy to improve efficacy, reduce toxicity, and personalize lung cancer treatment. Future studies should explore systemic immune responses and validate these findings in clinical settings to advance microbiome-guided cancer therapy.

## Data Availability

The data that support the findings of this study are available from the corresponding author upon reasonable request.

## Abbreviations

Lung cancer (LC); live bacterial therapeutic (LBT); lung adenocarcinoma (LUAD); squamous cell lung cancer (SCC); small cell lung cancer (SCLC); immune checkpoint inhibitor (ICI); antibiotic cocktail (Abx); short-chain fatty acid (SCFA); Lewis Lung Carcinoma (LLC); fecal microbiota transplant (FMT); exopolysaccharide (EPS); Food and Drug Administration (FDA).

## References

[1] Lahiri A., Maji A., Potdar P.D., Singh N., Parikh P., Bisht B., Mukherjee A., Paul M.K. (2023). Lung Cancer Immunotherapy: Progress, Pitfalls, and Promises. Mol. Cancer.

[2] Siegel R.L., Miller K.D., Jemal A. (2019). Cancer Statistics, 2019. CA Cancer J. Clin..

[3] Zeng J., Yi B., Chang R., Li J., Zhu J., Yu Z., Li X., Gao Y. (2024). The Causal Effect of Gut Microbiota and Plasma Metabolome on Lung Cancer and the Heterogeneity across Subtypes: A Mendelian Randomization Study. J. Pers. Med..

[4] Ryan A.M., Power D.G., Daly L., Cushen S.J., Ní Bhuachalla Ē., Prado C.M. (2016). Cancer-Associated Malnutrition, Cachexia and Sarcopenia: The Skeleton in the Hospital Closet 40 Years Later. Proc. Nutr. Soc..

[5] Bossi P., Delrio P., Mascheroni A., Zanetti M. (2021). The Spectrum of Malnutrition/Cachexia/Sarcopenia in Oncology According to Different Cancer Types and Settings: A Narrative Review. Nutrients.

[6] Prado C.M., Laviano A., Gillis C., Sung A.D., Gardner M., Yalcin S., Dixon S., Newman S.M., Bastasch M.D., Sauer A.C. (2022). Examining Guidelines and New Evidence in Oncology Nutrition: A Position Paper on Gaps and Opportunities in Multimodal Approaches to Improve Patient Care. Support. Care Cancer.

[7] Ren S., Feng L., Liu H., Mao Y., Yu Z. (2024). Gut Microbiome Affects the Response to Immunotherapy in Non-Small Cell Lung Cancer. Thorac. Cancer.

[8] Roy S., Trinchieri G. (2017). Microbiota: A Key Orchestrator of Cancer Therapy. Nat. Rev. Cancer.

[9] Zhang C., Wang J., Sun Z., Cao Y., Mu Z., Ji X. (2021). Commensal Microbiota Contributes to Predicting the Response to Immune Checkpoint Inhibitors in Non-Small-Cell Lung Cancer Patients. Cancer Sci..

[10] Zhou C.-B., Zhou Y.-L., Fang J.-Y. (2021). Gut Microbiota in Cancer Immune Response and Immunotherapy. Trends Cancer.

[11] Liu F., Li J., Guan Y., Lou Y., Chen H., Xu M., Deng D., Chen J., Ni B., Zhao L. (2019). Dysbiosis of the Gut Microbiome Is Associated with Tumor Biomarkers in Lung Cancer. Int. J. Biol. Sci..

[12] Xia L., Zhu X., Wang Y., Lu S. (2024). The Gut Microbiota Improves the Efficacy of Immune-Checkpoint Inhibitor Immunotherapy against Tumors: From Association to Cause and Effect. Cancer Lett..

[13] Zhu X., Li K., Liu G., Wu R., Zhang Y., Wang S., Xu M., Lu L., Li P. (2023). Microbial Metabolite Butyrate Promotes Anti-PD-1 Antitumor Efficacy by Modulating T Cell Receptor Signaling of Cytotoxic CD8 T Cell. Gut Microbes.

[14] Routy B., Le Chatelier E., Derosa L., Duong C.P.M., Alou M.T., Daillère R., Fluckiger A., Messaoudene M., Rauber C., Roberti M.P. (2018). Gut Microbiome Influences Efficacy of PD-1-Based Immunotherapy against Epithelial Tumors. Science.

[15] Bum Lee J., Huang Y., Oya Y., Nutzinger J., Ang Y.L.E., Sooi K., Cho B.C., Soo R.A. (2024). Modulating the Gut Microbiome in Non-Small Cell Lung Cancer: Challenges and Opportunities. Lung Cancer.

[16] Ting N.L.-N., Lau H.C.-H., Yu J. (2022). Cancer Pharmacomicrobiomics: Targeting Microbiota to Optimise Cancer Therapy Outcomes. Gut.

[17] Zhang M., Liu J., Xia Q. (2023). Role of Gut Microbiome in Cancer Immunotherapy: From Predictive Biomarker to Therapeutic Target. Exp. Hematol. Oncol..

[18] Subhadra B. (2019). Devices, Systems and Methods for the Production of Humanized Gut Commensal Microbiota. U.S. Patent.

[19] Subhadra B. (2018). Devices, Systems and Methods for the Production of Humanized Gut Commensal Microbiota. U.S. Patent.

[20] Subhadra B. (2020). Devices, Systems and Methods for the Production of Humanized Gut Commensal Microbiota. U.S. Patent.

[21] Subhadra B. (2024). Devices, Systems and Methods for the Production of Humanized Gut Commensal Microbiota. European Patent.

[22] Vivekanandan V., Khan Z.H., Venugopal G., Musunuru B., Mishra P., Srivastava S., Ramadass B., Subhadra B. (2024). VagiBIOM Lactobacillus Suppository Improves Vaginal Health Index in Perimenopausal Women with Bacterial Vaginosis: A Randomized Control Trial. Sci. Rep..

[23] Vakadaris G., Stefanis C., Giorgi E., Brouvalis M., Voidarou C.C., Kourkoutas Y., Tsigalou C., Bezirtzoglou E. (2023). The Role of Probiotics in Inducing and Maintaining Remission in Crohn’s Disease and Ulcerative Colitis: A Systematic Review of the Literature. Biomedicines.

[24] den Besten G., van Eunen K., Groen A.K., Venema K., Reijngoud D.-J., Bakker B.M. (2013). The Role of Short-Chain Fatty Acids in the Interplay between Diet, Gut Microbiota, and Host Energy Metabolism. J. Lipid Res..

[25] Alsholi D.M., Yacoub G.S., Rehman A.U., Ullah H., Khan A.I., Deng T., Siddiqui N.Z., Alioui Y., Farooqui N.A., Elkharti M. (2023). *Lactobacillus Rhamnosus* Attenuates Cisplatin-Induced Intestinal Mucositis in Mice via Modulating the Gut Microbiota and Improving Intestinal Inflammation. Pathogens.

[26] Bowen J.M., Stringer A.M., Gibson R.J., Yeoh A.S.J., Hannam S., Keefe D.M.K. (2007). VSL#3 Probiotic Treatment Reduces Chemotherapy-Induced Diarrhea and Weight Loss. Cancer Biol. Ther..

[27] Zhu S.-L., Ma Y.-Q., Zhu Z.-Y., Li Z.-M., Kang C.-Y., Chen H.-L., Chen P.-R., Han B., Yu T., Wang L.-S. (2021). Pectin Supplement Significantly Enhanced the Anti-PD-1 Efficacy in Tumor-Bearing Mice Humanized with Gut Microbiota from Patients with Colorectal Cancer. Theranostics.

[28] Levy B.D., Clish C.B., Schmidt B., Gronert K., Serhan C.N. (2001). Lipid Mediator Class Switching during Acute Inflammation: Signals in Resolution. Nat. Immunol..

